# Identification and expression analysis of *WRKY *transcription factor genes in canola *(Brassica napus *L.) in response to fungal pathogens and hormone treatments

**DOI:** 10.1186/1471-2229-9-68

**Published:** 2009-06-03

**Authors:** Bo Yang, Yuanqing Jiang, Muhammad H Rahman, Michael K Deyholos, Nat NV Kav

**Affiliations:** 1 Department of Agricultural, Food and Nutritional Science, Edmonton, Alberta T6G 2P5, Canada; 2 Department of Biological Sciences, University of Alberta, Edmonton, Alberta T6G 2E9, Canada

## Abstract

**Background:**

Members of plant WRKY transcription factor families are widely implicated in defense responses and various other physiological processes. For canola (*Brassica napus *L.), no WRKY genes have been described in detail. Because of the economic importance of this crop, and its evolutionary relationship to *Arabidopsis thaliana*, we sought to characterize a subset of canola *WRKY *genes in the context of pathogen and hormone responses.

**Results:**

In this study, we identified 46 *WRKY *genes from canola by mining the expressed sequence tag (EST) database and cloned cDNA sequences of 38 *BnWRKY*s. A phylogenetic tree was constructed using the conserved WRKY domain amino acid sequences, which demonstrated that BnWRKYs can be divided into three major groups. We further compared *BnWRKYs *to the 72 *WRKY *genes from *Arabidopsis *and 91 *WRKY *from rice, and we identified 46 presumptive orthologs of *AtWRKY *genes. We examined the subcellular localization of four BnWRKY proteins using green fluorescent protein (GFP) and we observed the fluorescent green signals in the nucleus only.

The responses of 16 selected *BnWRKY *genes to two fungal pathogens, *Sclerotinia sclerotiorum *and *Alternaria brassicae*, were analyzed by quantitative real time-PCR (qRT-PCR). Transcript abundance of 13 *BnWRKY *genes changed significantly following pathogen challenge: transcripts of 10 *WRKY*s increased in abundance, two *WRKY *transcripts decreased after infection, and one decreased at 12 h post-infection but increased later on (72 h). We also observed that transcript abundance of 13/16 *BnWRKY *genes was responsive to one or more hormones, including abscisic acid (ABA), and cytokinin (6-benzylaminopurine, BAP) and the defense signaling molecules jasmonic acid (JA), salicylic acid (SA), and ethylene (ET). We compared these transcript expression patterns to those previously described for presumptive orthologs of these genes in *Arabidopsis *and rice, and observed both similarities and differences in expression patterns.

**Conclusion:**

We identified a set of 13 *BnWRKY *genes from among 16 *BnWRKY *genes assayed, that are responsive to both fungal pathogens and hormone treatments, suggesting shared signaling mechanisms for these responses. This study suggests that a large number of BnWRKY proteins are involved in the transcriptional regulation of defense-related genes in response to fungal pathogens and hormone stimuli.

## Background

Canola (*Brassica napus*) is an economically important crop in Canada and other temperate regions, and is susceptible to adverse effects by fungal pathogens. Among these, *Sclerotinia sclerotiorum*, causing stem rot and *Alternaria brassicae*, causing Alternaria black spot, have potential to cause significant crop losses [1]. Considerable efforts are underway to develop canola varieties that are better able to tolerate these pathogens. We have previously used proteomics and genomics to survey the global changes in gene expression that occur as a result of pathogen challenge in canola [2-5].

Plant defense responses include the transcriptional control of expression of stress-responsive genes [6-9], including a number of transcription factors (TFs) whose abundance is altered as a result of the pathogen challenge. These TFs are presumably involved in regulating the expression of defense-related genes, and specifically include those containing Ethylene Response Factor (ERF)/Apetala2 (AP2)-domain, homeodomain, basic Leucine Zipper (bZIP), MYB, WRKY families and other zinc-finger factors, all of which have been observed to increase in response to pathogen challenge [10]. These defense-associated TFs can regulate downstream defense-related genes, and may themselves be regulated by phosphorylation [11-14].

The name of the WRKY family itself is derived from the most prominent feature of these proteins, the WRKY domain, which constitutes 60 amino acids [11]. In this WRKY domain, a conserved WRKYGQK heptapeptide is followed by a C_2_H_2_- or C_2_HC-type of zinc finger motif [11]. One or two WRKY zinc-finger motifs may be present, which can bind to the W-box DNA motif (C/T)TGAC(C/T) [15-19]. Furthermore, *cis*-elements other than TTGAC(C/T) have also been identified as a target of the WRKY domain of a barley WRKY TF [20,21]. The Group I WRKY TFs contain two WRKY domains: the C-terminal domain that plays a major role in binding to the W-box, while the N-terminal WRKY domain affects the binding affinity [15,16].

WRKY proteins belong to a super-family of zinc finger proteins [WRKY-Glial Cell Missing (GCM1)] containing six members [22]. For example, genes coding WRKY proteins were found not only in plants but also in the slime mold *Dictyostelium discoideum *and the protist *Giardia lamblia*, which indicates that WRKYs may have evolved prior to the evolution of plant phyla [23-25]. Some WRKY functions are thought to be conserved between phylogenetically distant species [26].

*WRKY *TF genes form large families in plants, with 72 members in *Arabidopsis *and close to 100 in *Oryza sativa *(rice) [27]. Previous studies have demonstrated that *WRKY *TFs are implicated in plant defense responses [14], sugar signaling [21] and chromatin remodeling [28]. Furthermore, *WRKY*s have been found to play essential roles in various normal physiological processes, including embryogenesis, seed coat and trichome development, senescence, regulation of biosynthetic pathways, and hormonal signaling [29-34]. As alluded to earlier, abiotic and biotic stresses are among the major external factors influencing the expression of *WRKY *genes in plants [11,23,35-38] and have been demonstrated to be involved in the defense against phytopathogens such as bacteria [25,39-42]; fungi [43-45]; and viruses [46,47].

The responses of *Arabidopsis *to pathogens have been observed to be mediated by signaling pathways [48-50]. For example, salicylic acid (SA) plays a positive role in plants against biotrophic pathogens, whereas jasmonic acid/ethylene (JA/ET) appears to be important in the case of necrotrophic pathogens [50-53]. It is also known that these (SA and JA/ET) signaling pathways are mutually antagonistic [54]. In *Arabidopsis*, it was observed that 49 out of 72 *AtWRKY *genes are regulated by *Pseudomonas syringae *or SA treatment [42]. On the other hand, of JA-responsive TF in *Arabidopsis*, AtWRKY TFs are one of the greatest numbers of induced [45]. Moreover, it is observed that cross-talk of SA- and JA-dependent defense response could be mediated by AtWRKY70, which is downstream of nonexpressor of pathogenesis-related gene 1 (*NPR1*) [55].

Previous studies have shown that abscisic acid (ABA), a negative factor in the SA and JA/ET signaling defense response, did not increase disease resistance [56-60]. However, recent research has demonstrated that ABA has a positive effect on callose deposition, which could lead to increased resistance of plants towards some pathogens [61-63]. Although WRKY TFs have been demonstrated to be involved in abiotic stress and ABA signaling [31,35,64-66], there are no reports available on the role of WRKYs in ABA-mediated biotic stress responses. The role of other hormones, such as cytokinins, has been investigated by many groups and it was observed that cytokinins, serving as endogenous inducers for distinct classes of pathogenesis-related (PR) proteins, are necessary for the biosynthesis of SA and JA [67-69]. Others have observed that the effect of cytokinins is mediated through the stimulation of ET production [70]. However, whether cytokinins induce the expression of PR genes through WRKYs is not presently clear.

Despite the obvious importance of WRKYs in responses to pathogens and hormone signaling, there are no reports as of yet, describing *WRKY *TFs in canola and their role(s) in mediating responses to pathogens. In our previous microarray analysis of canola response to *S. sclerotiorum*, we identified three WRKY genes whose transcript abundance was significantly affected by this fungus [5]. These results prompted us to systematically identify and examine *WRKY *TF genes in canola using the large set of available expressed sequence tags (ESTs). In this study, we analyzed ESTs from publicly available sequence information of canola and identified 46 sequences with similarities to *Arabidopsis WRKY *TFs. We investigated the evolutionary relationship of canola WRKY TFs with their counterparts from *Arabidopsis *and rice. We examined the subcellular localization of four BnWRKY proteins using green fluorescent protein (GFP). Subsequently, we studied the responses of representative members of monophyletically distinct WRKY clades to two fungal pathogens, as well as five plant hormones, in order to gain further insights into their roles in canola defense responses.

## Results

### Identification of 46 WRKY transcription factor genes in *B. napus*

Although the complete sequence of the *B. napus *genome has not yet been determined, the number of publicly available ESTs was 593,895 as of May 30, 2008. It is well known that gene discovery and genome characterization through the generation of ESTs is one of the most widely used methods [71]. A keyword search in NCBI "nr" dataset, returned only two previously annotated *BnWRKY *sequences. We used BLAST alignments to search the dbEST database and identified 343 unique GenBank EST accessions from *B. napus *that showed significant similarity to the 72 *AtWRKY *genes and 36 other *WRKY *sequences. We then used ESTpass to remove four chimerical ESTs and clustered the remaining 339 ESTs into 69 contigs and 66 singlets. For subsequent analyses, we also identified the largest open reading frame of each of the 135 contigs or singlets using OrfPredictor [72,73]. We also searched the DFCI oilseed rape gene index (BnGI, release 3.1) and identified 70 tentative consensuses (TC) and 79 singlets, which consisted of 314 ESTs. We found that all 314 of the BnGI ESTs were present within the 339 dbESTs we extracted from Genbank. The differences in numbers of WRKY ESTs from these databases can be explained by the fact that the number of entries in these databases are different, based on their release frequency. The Shanghai database , [74]) is more recent, with a greater number of entries, and produced an additional number of WRKY EST's that were incorporated in the current study. We note that the EST information available for canola is biased towards seed coat and embryo tissues, which likely limited our ability to identify a complete set of WRKY genes for this species. As the contigs/singlets output from ESTpass were annotated based on their similarity to *Arabidopsis WRKY *genes, we were able to identify the presumptive orthologs of the respective canola *WRKY *genes. Therefore, we assigned names to each *BnWRKY *(Additional file 1) based on the name of the corresponding *Arabidopsis WRKY*s.

We noted that among all the *BnWRKY *genes we annotated, *BnWRKY11 *has the largest number (40) of ESTs, followed by *BnWRKY32 *with a total of 26 ESTs, while *BnWRKY*26, 30, 36, and 66 have only one EST each (Additional file 1 and additional file 2). To facilitate subsequent phylogenetic, GFP fusion, and qRT-PCR analyses, we designed primers based on the identified ESTs for each of the 46 *BnWRKY *genes to obtain full length cDNA sequences, at least for each of the coding regions, employing RT-PCR together with 3'RACE. As a result, we succeeded in cloning the cDNA sequences of 38 of these 46 *BnWRKY *genes, among which we identified two different alleles (or possibly homeoalleles) for each of 13 *BnWRKY *genes (Additional file 1). We were also able to identify putative orthologs of these *BnWRKY *genes in both *Arabidopsis *and rice using the program InParanoid [75] (Additional file 1).

Although WRKY proteins have a conserved heptapeptide WRKYGQK motif [11], many studies have reported slight variations of the sequence for some WRKY proteins in *Arabidopsis*, rice, tobacco and barley [24,26,31,76]. Similarly, a number of the BnWRKYs we identified have amino acid sequence substitutions in their conserved WRKY signatures. For example, the following variations were noted: WRKYGKK in BnWRKY50, and WRKYGRK in BnWRKY51 (Additional file 3). We also observed a 25 amino acid insertion in the C-terminal WRKY domain of BnWRKY26, compared to AtWRKY26 (Additional file 3). An examination of the cDNA sequence of *Bn WRYKY26 *revealed that the insert starts with TT and ends with GG, suggesting that it is most probably not an intron. Usually the nucleotide sequence of the predominant class of introns begins with GT ends with AG and that of a minor class begins with AT and ends with AC [77,78], neither of which are true in this particular instance. Our results thus suggest that BnWRKY26 has diverged considerably during the evolutionary process.

### Phylogenetic analysis of BnWRKY proteins

**Figure 1:**
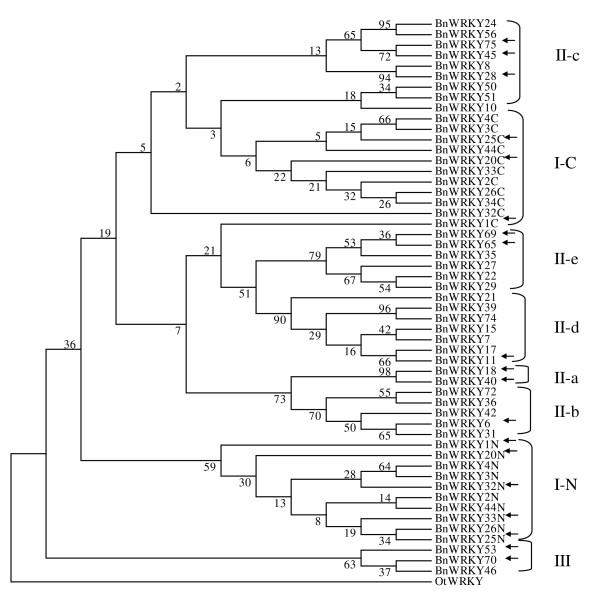
**A bootstrap consensus maximum parsimony tree of WRKY TFs in canola**. The phylogenetic tree was based on the amino acid sequences from WRKY domains only. Only the ~60 amino acid residues in the WRKY domain were aligned using ClustalX (v1.83) and were further examined manually for optimal alignment. The parsimony tree was drawn using MEGA4. The percentage of replicate trees is shown on the branches and it is calculated in the bootstrap test (500 replicates) for the associated taxa being clustered together. The two letters N and C after group I represent the N-terminal and the C-terminal WRKY domains of group I proteins, respectively.

From the 46 canola WRKY genes identified, we were able to extract 53 WRKY domains that were each approximately 60 amino acids in length. In 11 BnWRKY TF proteins, we identified two separate WRKY domains (Additional file 3), and both N- and C-terminal WRKY domains of these proteins were included in the phylogenetic analysis. The WRKY domain amino acid sequences were aligned with each other (Additional file 3) and a consensus maximum parsimony (MP) tree was inferred (Figure 1). Subsequently, we reconstructed a rooted MP tree using a WRKY protein from the world's smallest unicellular green algae *Ostreococcus tauri *WRKY as the outgroup (Figure 1). This tree demonstrates the polyphyletic nature of BnWRKY TFs, which is consistent with previous studies [22,23,26].

We next classified the BnWRKY TFs we identified into three major groups using criteria that had been previously described for this family [11]. Accordingly, the Group II proteins were further divided into five subgroups. From our study, at least two representatives for each subgroup of WRKY proteins were identified in the canola genome (Figure 1). For example, twelve *BnWRKYs *(*BnWRKY1*, 2, 3, 4, 19, 20, 25, 26, 32, 33, 34 and 44) code for proteins with two WRKY domains and clearly cluster with Group I of the AtWRKYs. The N- and C-terminal domains of these twelve BnWRKY form two different clusters named Group IN and Group IC (Figure 1). The 28 identified Group II WRKY members of canola were distributed unevenly among the five subgroups (subgroups IIa-e, Figure 1) and this is in agreement with previous studies in *Arabidopsis*, rice and barley [11,26,31]. Two BnWRKYs (BnWRKY18, 40) formed a distinct subclade, IIa, similar to the observations in *A. thaliana *[11]. Five canola WRKYs (BnWRKY6, 31, 36, 42, 72) belong to Group IIb; eight (BnWRKY8, 24, 28, 45, 50, 51, 56, 75) belong to Group IIc; seven (BnWRKY7, 11, 15, 17, 21, 39, 74) belong to Group IId; and six canola WRKY (BnWRKY22, 27, 29, 35, 65, 69) belong to Group IIe. Group III is represented by four single WRKY domain canola proteins (BnWRKY46, 53, 66 and 70). The comparison of number of WRKY genes in *Arabidopsis *(AtWRKY), rice (OsWRKY), barley (HvWRKY) and canola (BnWRKY) within each of the WRKY group/subgroups (Table 1) showed that about 53–59% of the expected WRKY genes of canola have been identified. It appears that within group IId, the same number of *WRKY *genes from *A. thaliana *and canola have been identified whereas for other subgroups, additional *BnWRKY *genes remain to be identified (Table 1). Our observations are similar to the study on the barley WRKY gene family in which approximately 50% of the expected *HvWRKY *genes were identified [26].

**Table 1:** Comparison of number of WRKY proteins of Arabidopsis (AtWRKY), rice (OsWRKY), barley (HvWRKY) and canola (BnWRKY) in each of the WRKY group/subgroups.

WRKY group	AtWRKY^a^	OsWRKY^b^	HvWRKY^c^	BnWRKY
I	15	13	8	12
IIa	3	4	4	2
IIb	8	7	1	5
IIc	17	20	11	8
IId	7	6	5	7
IIe	8	8	3	6
III	14	32	13	3
IV		6		

Total	72	96	45	43

According to a) Eulgem et al. [11], b) Xie et al. [31], Ross et al., [27], and c) Mangelsen et al. [26].

To further explore the phylogenetic relationships between *WRKY*s from canola and other species, we generated a phylogenetic tree incorporating all the *WRKY*s we identified from *Arabidopsis*, rice, and canola (Additional file 4; [27]). These results are consistent with our proposed classification of the newly characterized *WRKY*s from canola. However, in rice, there are four major groups of *WRKY*s, I, II, III and IV [27,31] and it can be observed that some members of the rice WRKY family are scattered throughout the phylogenetic tree, an observation that has also been made previously [27,31]. For example, OsWRKY57 (a group II WRKY) is clustered with those from group I-N and OsWRKY61 (a group Ib WRKY) is clustered with those of group III members (Additional file 4). Similarly, OsWRKY 9 and 83 (group Ia WRKYs) are clustered with group II members (groups IIb and d, respectively; Additional file 4). The three group IVa WRKYs (OsWRKY52, 56, and 58; Additional file 4) are scattered within branches of group II and III. Interestingly, we observed that OsWRKY86 (a group I member) is clustered with group III instead of group II as previously reported by others [27] and, OsWRKY84 is clustered within group III in our study, contrary to a previous report of this WRKY being clustered within group I ([38]; additional file 4). These discrepancies may be due to the use of different algorithms (neighbor-joining versus MP) to generate the phylogenetic trees.

### Nuclear localization of four BnWRKY proteins

**Figure 2:**
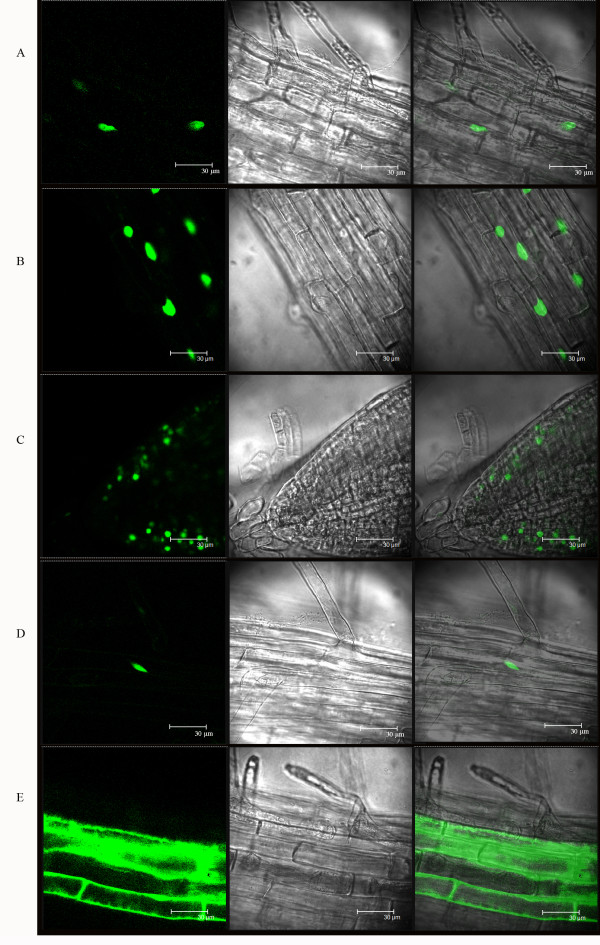
**Nuclear localization of four BnWRKY proteins**. Transgenic (T_2_) Arabidopsis roots of five-day old seedlings were observed under confocal microscope. Panels A-E represent the subcellular localization of BnWRKY6-sGFP, BnWRKY25-sGFP, BnWRKY33-sGFP, BnWRKY75-sGFP and pCsGFPBT vector control, respectively. In each case, the extreme left panel is GFP fluorescence, the middle bright field and the right represents an overlay of the two images.

The function of a TF normally requires that it is localized in the nucleus, although TFs targeting chloroplasts, mitochondria, or endoplasmic reticulum (ER) have also been identified [79]. To confirm that the BnWRKY TFs we identified are indeed targeted to the nucleus, we selected four *BnWRKY *genes based on their known functions in mediating defense responses in *Arabidopsis *[25,45,80-82] for analysis *in vivo*. We fused the coding regions of *BnWRKY6*, 25, 33, and 75 to the N-terminus of synthetic green fluorescent protein (sGFP) [83] and expressed them in *Arabidopsis *under the control of the constitutive cauliflower mosaic virus (CaMV) 35*S *promoter. Analysis of conceptually translated *BnWRKY6*, 25, and 33 coding sequences revealed the presence of a monopartite nuclear localization signal (NLS) (prediction program of protein localization sites, ), however, no NLS was detected in the translated BnWRKY75 sequence. We analyzed transgenic *Arabidopsis *seedlings harboring the respective four constructs. In all four cases, green fluorescent signals were observed only in the nucleus (Figure 2A–D). With the control vector alone, GFP signals were distributed in both the cytoplasm and nucleus (Figure 2E). Our results indicate that *BnWRKY6, 25*, 33, and 75 are indeed nuclear-localized proteins, which is consistent with their predicted function as transcription factors.

### Expression analysis of *BnWRKY *genes in response to fungal pathogens-*S. sclerotiorum *and *A. brassicae*

**Figure 3:**
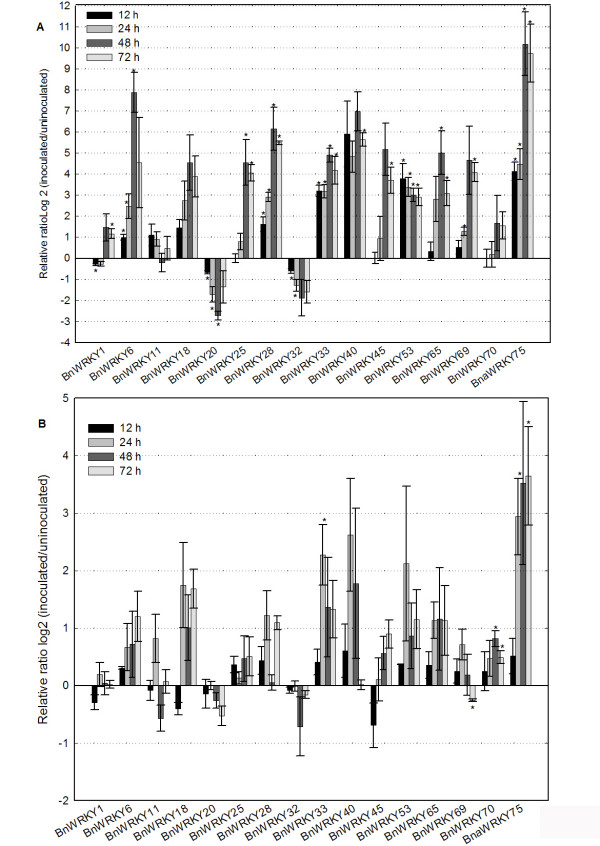
**Expression analyses of *BnWRKY *genes in response to fungal challenge**. Changes in *BnWRKY *transcript abundance in response to (A) *S. sclerotiorum *and (B) *A. brassicae *infection. Data is the mean of three biological replicates ± S.E.

Because the divergence of paralogous genes is often after the sub-functionalization [84], we employed qRT-PCR to investigate the responses of representatives of each of the three major *WRKY *clades. We selected 16 *BnWRKY *genes, *WRKY1*, 6, 11, 18, 20, 25, 28, 32, 33, 40, 45, 53, 65, 69, 70 and 75, as representatives of each clade (Additional file 1, Figure 1). After challenge with the fungal pathogen *S. sclerotiorum*, transcript abundance of 13 *BnWRKY *genes was observed to be significantly (t-test, P < 0.05) modulated with 10 being increased, two being decreased and one being decreased at 12 h but subsequently increased at 72 h (Figure 3A). *BnWRKY6*, 25, 28, 33, 40, 45, 53,65, 69 and75 were highly induced at 48 h after the inoculation. However, *BnWRKY20 *and 32 were repressed by *S. sclerotiorum *infection. *BnWRKY1 *was observed to be repressed at an earlier time point (12 h) but induced later (72 h, Figure 3A).

We then examined the changes in transcript abundance of these 16 *BnWRKY *genes in response to a second fungal pathogen, *A. brassicae*, which is also a necrotrophic pathogen. The symptom development in these two pathosystems (*S. sclerotiorum *and *A. brassicae*) is different with respect to time required, with *A. brassicae *requiring a much longer period before visible disease symptoms could be observed (data not shown). Accordingly, the transcript abundance of only four *BnWRKY *genes were significantly affected by *A. brassicae *with two (*BnWRKY33 *and 75) being significantly increased at 48 h post-pathogen challenge and two (*BnWRKY70 *at both 48 and 72 h and *BnWRKY69 *only at 72 h) with decreased transcript abundance (Figure 3B). In summary, our results indicate that *BnWRKY33 *and 75 are induced by both *S. sclerotiorum *and *A. brassicae *with *BnWRKY75 *exhibiting a similar temporal pattern of changes in transcript abundance between the two fungi. However, *BnWRKY69 *and 70 had different responses to *S. sclerotiorum *and *A. brassicae*. Our results suggest that although both pathogens investigated in this study are necrotrophic, they elicit slightly different responses with respect to changes in transcript abundance of *BnWRKY *genes.

### Response of selected *BnWRKY *genes to hormone treatments

To investigate the hormonal control mechanisms underlying *BnWRKY *gene expression, we treated canola plants with five phytohormones, JA, SA, ABA, BAP and ET and analyzed the changes in transcript abundance of these 16 *BnWRKY *genes using qRT-PCR. To ensure that the hormone applications were eliciting expected responses in plants, we first examined the responses of a few additional canola genes that are proposed to be orthologs of *Arabidopsis *genes previously reported to respond to these hormones. These *Arabidopsis *genes were two *bZIP *transcription factors, *TGA5*, *TGA6 *for SA [85-87]; allene oxide cyclase (*AOC*) [88] and plant defensin 1.2 (*PDF1.2*) for JA [50]; ethylene insensitive 2 (*EIN2*) [89] and ethylene responsive factor (*ERF2 *and *ERF4*) [90] for ET; ABA insensitive 5 (*ABI5*) [91-93] for ABA, and *Arabidopsis *response regulator 6 (*ARR6*) [94] and cytokinin response 1 (*CRE*) [95] for BAP. We observed that the abundance of transcripts for all of these genes was significantly increased in response to the hormone treatments (data not shown), confirming the efficacy of our hormone treatments.

**Figure 4:**
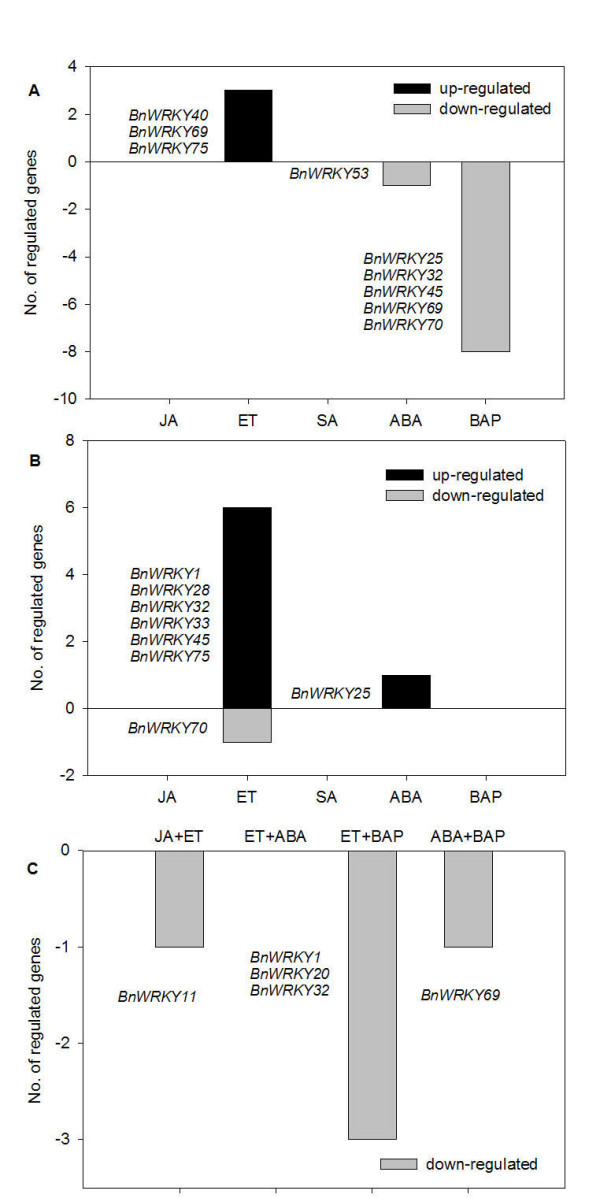
**Expression analyses of *BnWRKY *genes in response to different hormone treatments**. Changes in *BnWRKY *transcript abundance as a result of hormone application at (A) 6 h, (B) 24 h and (C) those that respond to more than one hormone at 6 h.

Our results demonstrated that among the 16 *BnWRKY *genes studied, *BnWRKY40*, 69 and 75 were induced by ET and *BnWRKY53 *was repressed by ABA at 6 h (Figure 4A, Table 2). In contrast, *BnWRKY25*, 32, 45, 69 and 70 were repressed by BAP at 6 h (Figure 4A, Table 2). At 24 h, *BnWRKY1*, 28, 32, 33, 45, and 75 were specifically induced by ET and *BnWRKY70 *was repressed by ET (Figure 4B, Table 2). Three *BnWRKY *genes exhibited modulation of expression in response to two hormones (Table 2). At 6 h, both JA and ET repressed *BnWRKY11 *and both ET and BAP repressed *BnWRKY1*, 20 and32 (Figure 4C, Table 2). However, none of the genes were observed to be affected by the two hormones at 24 h. In addition, both ABA and BAP repressed *BnWRKY69 *(Figure 4C, Table 2). None of these *BnWRKY *genes were affected by three or more hormones (Table 2).

**Table 2:** Expression analyses of *BnWRKY *genes to five plant defense-related hormone treatments assayed by qRT-PCR.

gene	JA		ET		SA		ABA		BAP	
	
	6 h	24 h	6 h	24 h	6 h	24 h	6 h	24 h	6 h	24 h
*BnWRKY1*	2.47 (± 1.40)	1.11 (± 0.06)	0.83 (± 0.03)*	1.13 (± 0.03)*	1.53 (± 0.26)	1.10 (± 0.2)	2.54 (± 1.31)	1.03 (± 0.08)	0.59 (± 0.02)**	0.58 (± 0.08)*
*BnWRKY6*	0.64 (± 0.14)	0.88 (± 0.29)	1.07 (± 0.59)	3.98 (± 0.81)	1.99 (± 0.39)	1.29 (± 0.37)	2.17 (± 0.17)	1.23 (± 0.13)	0.72 (± 0.13)	0.64 (± 0.22)
*BnWRKY11*	0.63 (± 0.01)**	0.85 (± 0.12)	0.70 (± 0.00)**	1.02 (± 0.10)	1.63 (± 0.21)	1.22 (± 0.29)	1.16 (± 0.03)	1.06 (± 0.37)	0.64 (± 0.11)	0.97 (± 0.05)
*BnWRKY18*	1.99 (± 0.64)	1.31 (± 0.33)	1.74 (± 0.20)	1.60 (± 0.33)	11.65 (± 4.02)	6.48 (± 0.59)	3.01 (± 0.62)	1.07 (± 0.39)	0.71 (± 0.27)	0.38 (± 0.11)*
*BnWRKY20*	0.85 (± 0.24)	0.95 (± 0.12)	0.68 (± 0.04)*	1.38 (± 0.13)	1.38 (± 0.35)	1.19 (± 0.15)	0.77 (± 0.15)	0.87 (± 0.03)	0.61 (± 0.07)*	0.59 (± 0.14)
*BnWRKY25*	1.71 (± 0.54)	1.53 (± 0.16)	1.82 (± 0.16)	2.34 (± 0.50)	1.69 (± 0.39)	0.92 (± 0.09)	2.51 (± 1.45)	1.83 (± 0.19)*	0.55 (± 0.07)*	0.42 (± 0.13)*
*BnWRKY28*	0.75 (± 0.11)	1.55 (± 0.25)	0.86 (± 0.26)	1.49 (± 0.05)**	1.37 (± 0.45)	3.64 (± 3.00)	1.00 (± 0.09)	1.75 (± 1.02)	1.00 (± 0.38)	1.32 (± 0.73)
*BnWRKY32*	1.11 (± 0.15)	1.14 (± 0.15)	0.81 (± 0.04)*	1.33 (± 0.06)*	1.44 (± 0.34)	0.88 (± 0.08)	1.27 (± 0.17)	0.97 (± 0.12)	0.73 (± 0.04)*	0.82 (± 0.14)
*BnWRKY33*	0.68 (± 0.30)	0.76 (± 0.09)	3.89 (± 0.09)	2.38 (± 0.31)*	4.80 (± 1.23)	1.40 (± 0.36)	0.50 (± 0.15)	1.00 (± 0.21)	1.09 (± 0.17)	0.87 (± 0.18)
*BnWRKY40*	0.84 (± 0.2)	1.17 (± 0.44)	4.74 (± 0.05)*	6.49 (± 1.63)	2.17 (± 0.47)	0.99 (± 0.24)	1.28 (± 0.47)	1.69 (± 0.74)	0.61 (± 0.05)*	0.56 (± 0.16)
*BnWRKY45*	1.5 (± 0.51)	0.91 (± 0.21)	1.29 (± 0.17)	3.41 (± 0.39)*	1.39 (± 0.17)	1.11 (± 0.20)	2.35 (± 1.07)	1.71 (± 0.21)	0.63 (± 0.01)**	0.94 (± 0.21)
*BnWRKY53*	0.39 (± 0.14)	0.89 (± 0.50)	2.14 (± 0.07)	0.75 (± 0.10)	8.14 (± 1.69)	2.33 (± 1.05)	0.45 (± 0.00)**	0.81 (± 0.37)	1.43 (± 0.19)	2.08 (± 0.69)
*BnWRKY65*	1.41 (± 0.36)	1.58 (± 0.51)	1.82 (± 0.70)	1.46 (± 0.33)	1.64 (± 0.40)	1.84 (± 0.71)	0.77 (± 0.19)	1.16 (± 0.42)	0.78 (± 0.17)	0.50 (± 0.05)**
*BnWRKY69*	0.71 (± 0.10)	1.04 (± 0.27)	1.29 (± 0.01)**	1.76 (± 0.29)	1.29 (± 0.16)	1.15 (± 0.19)	0.42 (± 0.08)*	1.17 (± 0.41)	0.65 (± 0.08)*	0.56 (± 0.10)*
*BnWRKY70*	0.84 (± 0.16)	1.12 (± 0.21)	1.43 (± 0.24)	0.52 (± 0.00)**	13.98 (± 6.01)	3.66 (± 2.00)	0.85 (± 0.12)	1.01 (± 0.36)	0.46 (± 0.04)**	0.83 (± 0.26)
*BnWRKY75*	1.55 (± 0.36)	2.30 (± 0.93)	2.39 (± 0.03)*	9.21 (± 0.63)*	17.58 (± 12.54)	8.19 (± 4.61)	4.53 (± 2.14)	2.03 (± 0.43)	0.50 (± 0.24)	0.60 (± 0.30)

Results are presented as a ratio of transcript abundance in treatment/mock on a linear scale. Data were mean of three biological replicates ± S.E. The asterisk indicates that the corresponding gene was significantly up- or down-regulated under a stress treatment by *t*-test (* for p < 0.05 and ** for p < 0.01).

As indicated earlier, JA and SA are important signaling molecules which are implicated in plant defense responses [96,97]; and other phytohormones, through their effect on SA or JA signaling, may influence disease outcomes [98]. *BnWRKY11 *was observed to be repressed by JA at 6 h although no significant change was observed at 24 h (Table 2). In response to SA treatment, we observed that the transcript abundance for seven genes (*BnWRKY6*, 18, 33, 40, 53, 70 and 75) exhibited modulation at 6 h and three (*BnWRKY53*, 70 and 75) at 24 h (Table 2), however, these observed changes were not statistically significant.

In summary, SA did not significantly affect the transcript abundance of any of the *BnWRKYs *tested, whereas ET, ABA, JA and the cytokinin BAP did affect the transcript abundance of various *BnWRKY *genes investigated in this study (Table 2). Although the 16 genes tested did not show significant changes in expression levels after exogenous treatments with SA, there is the possibility that other *BnWRKY *genes may be responsive to SA.

## Discussion

In this study, we describe the identification and annotation of cDNA sequences of 46 members of the *WRKY *gene family in canola and their classification into groups I to III (Figure 1, Additional file 1). Among the 46 *BnWRKY *genes identified, both the hallmark WRKYGQK motif (43) and its variants (two variants, WRKYGKK for BnWRKY50 and WRKYGRK for BnWRKY51) were identified in the translated amino acid sequences while that of BnWRKY10 waits to be identified (Additional file 3). A recent study demonstrated that AtWRKY TFs bearing the WRKYGQK motif exhibit binding site preferences, which are partly dependent on the adjacent DNA sequences outside of the TTGACY-core motif [20]. For those WRKY TFs that do not contain the canonical WRKYGQK motif, a binding sequence other than the W-box element ((C/T)TGAC(C/T)) may exist. For instance, the binding sequence of tobacco (*Nicotiana tabacum*) NtWRKY12 with a WRKYGKK motif is TTTTCCAC, which deviates significantly from W-box [76]. Moreover, soybean (*Glycine max*) GmWRKY6 and GmWRKY21 lose the ability to bind to a W-box containing the variant WRKYGKK motif [66]. It seems likely that the BnWRKY TFs that lack the canonical WRKYGQK motif might not be able to interact with W-box and therefore may have different target genes and possibly divergent roles, a proposal that must be verified in future studies. Finally, mutation of amino acid Q to K of AtWRKY1 was observed to affect binding activity with the consensus W-box [99]. Furthermore, the second characteristic feature of WRKY proteins is a unique zinc-finger motif C-X_4–5_-C-X_22–23_-H-X-H [11]. Of the 53 BnWRKY domains, most of them contain this unique zinc-finger motif while BnWRKY46, 53 and 70 (group III) have an extended zinc-finger motif which is C-X_7_-C-X_23_-H-X-C. This observation is consistent with previous study in *Arabidopsis *[11], barley [26] and rice [100].

Complete or partial WRKY domains are found in ESTs from many species of land plants [24]. Recently, 37 *WRKY *genes were identified in the moss, *Physcomitrella patens *[101]. So far, no *WRKY *genes have been identified in the archaea, eubacteria, fungi, or animal lineages [24]. However, in the genomes of the protist, *Giardia lamblia *and the slime mold, *Dictyostelium discoideum*, a single *WRKY *gene with two *WRKY *domains were recently identified [23,24]. Further examination of the two WRKY domains existing in the two organisms indicates that *G. lamblia *WRKY TF has a WRKYGSK heptapeptide at its N-terminal and a WKKYGHK at its C-terminal, whereas in *D. discoideum*, both WRKY domains have a classical WRKYGQK heptapeptide [24,101]. This suggests an ancient origin of the canonical WRKYGQK heptapeptide and its variants. In the green algae, *Chlamydomonas reinhardtii*, a WRKY TF containing two WRKY domains (Acc. XM_001692290) was also identified [24,101]. In the genome of the recently sequenced, world's smallest free-living eukaryote, the unicellular chlorophytic algae, *Ostreococcus tauri*, a WRKY gene containing a single WRKY domain and a WRKYGCK heptapeptide is also present (Acc. CAL54953).

The identification of WRKY genes in primitive eukaryotes suggests an ancient origin of the WRKY family, and this family had emerged before the evolution and diversification of the plant phyla [24]. During the long evolutionary history, the WRKY gene family greatly expanded, as demonstrated by the increased numbers of *WRKY *genes in higher plants [101], and this expansion may be primarily due to segmental duplications of genomic fragments as a result of independent polyploidy events [24,102-104]. Comparison of a genomic region harboring five genes, one of which is *WRKY10*, between tomato, *Arabidopsis *and *Capsella rubella*, has revealed a great degree of microsynteny between closely and distantly related dicotyledonous species [105]. In addition, AtWRKY10, with one WRKY domain, is clustered within group I AtWRKYs possessing two-domains [11]. The ortholog of AtWRKY10 in tomato or rice (OsWRKY35, Os04g39570) contains two WRKY domains, which may suggest that during evolution, the N-terminal domain has been lost and the occurrence of the loss of the N-terminal WRKY domain of AtWRKY10 is after the divergence of monocots and dicots [27,105].

An overall genomic duplication event has been identified to exist in the tribe *Brassiceae *after a comparative genomic analysis, and many genomic units that are conserved between canola and *Arabidopsis *have also been identified [106-108]. We observed Brassicaeceae-specific clades and rice-specific clades based on our current analysis. In group III, *Arabidopsis *WRKY domains (six AtWRKYs) form Brassicaeceae-specific clade while rice WRKY domains (23 OsWRKYs) also form rice-specific clade (Additional file 4). Similar to that observed by [26], some monocot-specific clades were observed in groups IIc and III WRKY domains of rice and barley. This further supports the conclusion by Mangelsen et al. [26] about the occurrence of this diversification after the divergence of mono- and di-cotyledonous plants. Possibly more Brassicaeceae-specific WRKY clades could be indentified from future phylogenetic analysis after the whole genome of canola has been determined. A further comparative genomic analysis of the *WRKY*-containing regions between canola and *Arabidopsis *should enable us to reveal the extent of microcolinearity between these closely related species and a better understanding of the expansion of the *WRKY *gene family in canola.

WRKY TFs are involved in the regulation of various biological processes, including pathogen responses and hormone signaling [109]. A previous expression analysis of *AtWRKY *genes demonstrated that nearly 70% are differentially expressed by pathogen infection or SA treatment, suggesting important roles for *WRKY *in defense responses [42]. Recently, two studies of the rice *WRKY *genes also demonstrated that many are responsive to JA, SA and ABA treatments [37,38]. Increased transcript abundance of SA- and JA-responsive genes is essential for the induced resistance conferred by the two signaling pathways [97,110,111]. *WRKY *TFs are also reported to participate in disease resistance in *Arabidopsis *and tobacco through modulation of SA- or JA-responsive gene expression similar to that induced by the TGA class of basic leucine-zipper transcription factors [39,40,45,81,112].

Previous studies from our laboratory as well as those of others revealed that few genes related to SA-signaling were modulated by infection of canola with *S. sclerotiorum *[2,5], suggesting that SA does not play a crucial role in mediating responses of canola to this pathogen. The responses of *Arabidopsis *to *A. brassicicola*, which causes black spot in canola as *A. brassicae *does, also appear to be mediated through JA instead of SA [113], which is similar to responses to other necrotrophic fungi, including *Pythium *species [80,114]. Hence, it is possible that WRKY TFs may play an important role in suppressing the involvement of SA in response to those pathogens. This suggestion is consistent with the conclusion that AtWRKY33, which is induced by many pathogens, acts as a positive regulator of JA- and ET-mediated defense signaling but as a negative regulator of SA-mediated responses [45]. As mentioned earlier, both *A. brassicae *as well as *S. sclerotiorum *are able to induce *BnWRKY33*, one of the genes belonging to group I. Moreover, it has been demonstrated that pathogen-induced *AtWRKY33 *expression does not require SA signaling [80]. Similar to *AtWRKY33*, *AtWRKY25 *acts as a negative regulator of the SA-mediated signaling pathway [25]. The increased abundance of *BnWRKY25 *due to the infection by *S. sclerotiorum*, but not in the case of *A. brassicae *challenge, also suggests that it might also work as a negative regulator of SA-related signaling pathways in the canola-*S. sclerotiorum *pathosystem, but not in the canola-*A. brassicae *pathosystem. Of the other group I members investigated in our study, (*BnWRKY1, 20 and 32*), *BnWRKY1 *was observed to be significantly induced by *S. sclerotiorum *only at 72 h (Figure 3), and *BnWRKY20 *and 32 were repressed by *S. sclerotiorum *(Figure 3), indicating the differences in behavior of group I BnWRKYs in response to fungal pathogens (*S. sclerotiorum *and *A. brassicae*).

**Figure 5:**
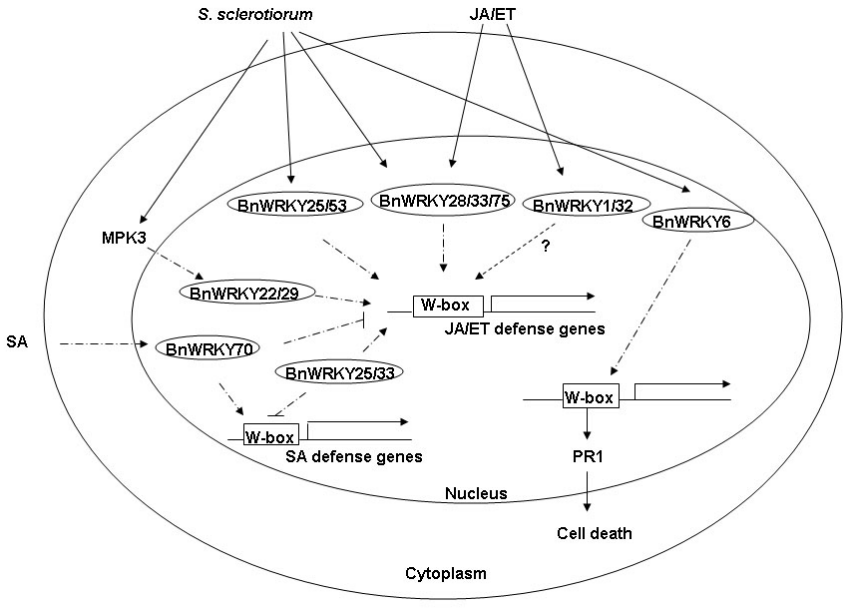
**Hypothetical model of WRKY network in mediating canola responses to *S. sclerotiorum *and phytohormones**. JA/ET-responsive, but not SA-responsive genes were observed in the necrotrophic pathogen *S. sclerotiorum*-canola interaction [2,5], and these induced *BnWRKY *genes, such as *BnWRKY25*, *28*, *33*, *53 *and *75*, could potentially activate the downstream JA/ET signaling pathway or cell death. Moreover, BnWRKY70 might also negatively regulate JA/ET signaling pathway while BnWRKY6 possibly modulates the expression of *PR1 *gene, culminating in cell death, which would benefit the infection and growth of necrotrophic fungi [114,118]. MPK3, one of mitogen-activated protein kinases, was also observed to be induced by *S. sclerotiorum *in our previous microarray study [5], and MPK3 in Arabidopsis is a positive regulator of AtWRKY22/29 [39]. Solid and dashed arrows represent likely or putatively positive regulation of the downstream targets while open blocks indicate negative regulation of the downstream genes.

It is possible that several BnWRKY TFs may also be involved in signaling the responses of canola to the pathogens *S. sclerotiorum *and *A. brassicae*. For instance, group IIa members have been demonstrated to play both positive and negative roles in plant defense [112,115-117]. The transcript levels of two genes of the group IIa: *BnWRKY18*, and 40, orthologs of which are known to act as negative regulators of plant defense in *Arabidopsis *[95], were observed to increase in response to *S. sclerotiorum *and *A. brassicae *challenge. For *A. brassicae*, the differences in transcript abundance between controls and inoculated plants were not statistically significant (Figure 3). In addition, it has been reported that *AtWRKY6*, one member of the group IIb, acts as a positive regulator of the senescence- and pathogen defense-associated *PR1 *promoter activity, and is also induced by SA and bacterial infection [114]. Since leaf senescence is often linked to plant defense [118], the induction of *BnWRKY6 *by *S. sclerotiorum*, ABA and SA at an early time-point (6 h) but not *A. brassicae*, may suggest a role in leaf senescence, which is observed very early in the *S. sclerotiorum*-canola pathosystem (Figure 5).

We also observed that group IIc (*BnWRKY2*8,45) and III (*BnWRKY75*) BnWRKYs in our study were all induced by the infection of *S. sclerotiorum *and ET whereas *BnWRKY75 *was induced only by *A. brassicae *(Figure 3, Table 2). Changes in expression of *BnWRKY75 *induced by both pathogens suggest that an ET-mediated signaling pathway may be involved in mediating the responses of canola to necrotrophic pathogens. In *Arabidopsis *growing in 1/2 × MS liquid media supplemented with 10 μM of 1-aminocyclopropane-1-carboxylate (ACC), it has been previously observed that *AtWRKY45 *and *AtWRKY75 *were induced; while *AtWRKY28 *was repressed compared to untreated controls [119]. This differences between the expression of *AtWRKY28 *[116] and *BnWRKY28 *(this study) in response to ET treatment may be the result of subtle differences in experiments including the use of different tissues (seedlings versus leaves), and/or the ethylene-generating reagents (ACC versus ET) used. Further studies on the role of these three BnWRKY TFs in mediating defense responses are ongoing in our laboratory.

Although *BnWRKY11 *(Group IId) was not affected by either *S. sclerotiorum *or *A. brassicae *in this study, our previous microarray profiling of transcriptome changes in canola as a result of *S. sclerotiorum *infection revealed that transcript levels of *BnWRKY11 *and 15 increased while that of *BnWRKY17 *decreased at specific time points, although the magnitude of response was less than two-fold [5]. *Arabidopsis AtWRKY11 *and *AtWRKY17 *are both known to act as negative defense regulators and *WRKY11 *appears to act upstream of JA [120] since it does not respond to JA [119,121]. However, the expression of *AtWRKY11 *has been reported to correlate with the induction of the JA biosynthesis enzymes AOS and LOX 2 h after challenge with *P. syringae *[120]. Incidentally, the accumulation of JA also occurs within the first hour of the interaction with *P. syringae *[122]. Taken together with our observations that *BnWRKY11 *was repressed by JA and ET treatments at 6 h, and was not induced by the pathogens, it is possible that the pathogen-induced accumulation of JA might modulate the expression of *BnWRKY11*.

Given the recently emerging role for ABA in defense responses [61-63], it is possible that ABA exerts some of these effects through the modulation of *BnWRKY *genes, specifically *BnWRKY53 *and *BnWRKY69*. Similarly, the cytokinin BAP has been implicated in both alleviating and exacerbating the hypersensitive response (HR), which is characterized by tissue necrosis and is frequently accompanied by the subsequent induction of systemic acquired resistance (SAR) throughout the plant [123]. Furthermore, cytokinins can promote the susceptibility of biotrophs by inducing the necrotroph resistance pathway, which is responsive to JA/ET [98]. As suggested for ABA mediated plant defense responses, it is possible that the BnWRKYs, which were observed to be modulated by exogenous BAP application, may be responsible, at least in part, for mediating the observed effects with the necrotrophic pathogens.

Based on our previous and current studies, we propose a model outlining the possible roles of BnWRKY TFs in mediating the responses of canola to *S. sclerotiorum *and phytohormones (Figure 5). Both *S. sclerotiorum *and JA/ET can induce *BnWRKY28*, 33, and 75 at 24 h post inoculation and possibly activate the downstream JA/ET signaling pathway at later time points (Figure 5). *S. sclerotiorum *specifically induces *BnWRKY6*, 25, and 53, and JA/ET specifically induce *BnWRKY1 *and 32 at 24 h post inoculation (Figure 5). This may be explained by the more complicated molecular patterns generated by *S. sclerotiorum *during the infection compared to the application of JA or ET only. BnWRKY70 in this study is also found to negatively regulate JA/ET defense genes, and it is possibly positively regulated by SA (Figure 5), which could lead to interference with the JA/ET signaling pathway. This is consistent with previous report of AtWRKY70 being the node of convergence of JA and SA signaling [55]. It is also possible that BnWRKY6 modulates the expression of *PR1 *gene, culminating in cell death (Figure 5), which is conducive for the growth of necrotrophic fungi [114,118], a hypothesis that must be tested in the future. A mitogen-activated protein kinase (MAPK) gene, *MPK3*, was also observed to be induced at 24 h and 48 h post inoculation in our previous study [5]. MPK3 in *Arabidopsis *has been demonstrated to positively regulate *AtWRKY22/29 *[39], (Figure 5). Similar to AtWRKY25 and 33, BnWRKY25 and 33, may act as a positive regulators of JA- and ET-mediated defense signaling pathways and as negative regulators of the SA-mediated signaling pathway [25,45] (Figure 5). Further work needs to be performed to characterize orthologs of AtWRKY22 and 29 in canola, and to examine the relationship between MAPK signaling cascade and BnWRKYs. Moreover, a detailed functional characterization of BnWRKY TFs, using a variety of reverse genetic techniques, in the context of *S. sclerotiorum *response, will help to better delineate their physiological roles.

As discussed above for *B. napus*-*S. sclerotiorum *pathosystem and function of related *AtWRKY *gene, several WRKY factors act as negative regulators of plant defense whereas others positively modulate this response implying their association with distinct regulatory complexes. Functional redundancy in defense programs is an inherent feature of WRKY genes [109] and it may reflect a strong need to backup essential regulatory functions [22]. Still, we can expect exciting novel revelations about WRKY TFs in the very near future on the basis of the enormous progress made within the past two years.

## Conclusion

In summary, we identified 46 BnWRKY TFs based on the publicly available EST resources of canola and cloned the cDNA sequences for 38 of them. We characterized the responses of 16 selected genes, based on their phylogenetic relationship in response to two fungal pathogens and five hormone treatments. Based on our data, we propose that BnWRKY TFs might play an important role in plant defense response, possibly by acting as positive or negative regulators of plant defense, and canola may respond differently to *S. sclerotiorum *and *A. brassicae *from BnWRKY mediated plant defense system. Our results also confirm that there is cross-talk between biotic stress and hormone signaling. Functional redundancy in defense programs is an inherent feature of WRKY genes [109] and future studies will be directed towards delineating the specific roles of individual WRKY TFs in those and related pathosystems in order to explore the possibility that manipulation of abundance of one or several of these proteins may lead to durable and robust resistance to the pathogen, apart from contributing to our understanding of the molecular processes that occur during host-pathogen interactions.

## Methods

### *BnWRKY *gene identification

Thirty-six WRKY domain sequences (WRKY-seed) downloaded from Pfam  were used to search the dbEST  datasets (release 053008) for WRKY genes in *B. napus *(oilseed rape and canola) using the tBlastn program. The significant hits (E < 1e-4) were retrieved and Microsoft Excel 2003 was then used to obtain unique sequences based on the GenBank Accession numbers. 177 unique ESTs were retrieved and organized into a FASTA format file before input into ESTpass program [124] for cleansing, clustering, and assembling of the unique ESTs. To confirm that the obtained contigs and singlets encode WRKY proteins, the nucleotide sequences were translated in six possible reading frames using OrfPredictor into amino acid sequences, which were then examined for the existence of the heptapeptide WRKYGQK and its variants. The resulting 36 contigs and 38 singlets were used as query sequences in a BLASTn search against *B. napus *EST dataset in NCBI dbEST and Shanghai RAPESEED database (, [74]) in order to obtain maximum sequence length for each *BnWRKY*, and 339 unique ESTs were retrieved. We also used a key word search of *WRKY *genes in *B. napus *in the non-redundant (nr) database of NCBI and obtained two cDNA sequences (GenBank Acc. DQ539648 and DQ209287), which were annotated to be *BnWRKY40*. Altogether we obtained 341 unique sequences based on the accession numbers. We then used the ESTpass program for cleansing, clustering, and assembling of the unique ESTs. The resultant contigs and singletons were then used as query sequences in a Blastn search against *Arabidopsis *to find the best hit (putative orthologs) among the 72 *AtWRKY *genes. Afterwards, the putative transcripts were analyzed using OrfPredictor to predict open reading frames (ORFs) and obtain the translated amino acid sequences. The amino acid sequence of the largest ORF for each putative transcript was filtered out and entered into the SMART program  to predict the WRKY domain. In case of the absence of the characteristic features of the WRKY domain for a particular transcript, it was translated in six possible reading frames in DNAMAN (V4.0, Lynnon BioSoft) and manually checked to output the amino acid sequences. At this step, we obtained 46 unique *BnWRKY *genes and identified those *BnWRKY *genes that contain incomplete or no WRKY domain and therefore we used RT-PCR together with 3'RACE to extend the WRKY domain sequences.

### Plant growth and gene cloning

Wild type canola (Westar) plants were grown in Sunshine soil mix 4 (Sungro, Vancouver, BC, Canada) in the greenhouse with a photoperiod of 16 h light (combination of natural light and T5 fluorescent tubes with a light intensity of approximately 200 μE m^-2 ^s^-1^)/8 h dark, and a temperature of 21°C day/18°C night for 18 days. Young leaves were harvested for RNA isolation using the RNeasy Plant Mini kit (Qiagen, Mississauga, ON, Canada). RNA integrity was checked by electrophoresis on a formaldehyde agarose gel and quantified using the NanoDrop 1000 (NanoDrop Technologies, Inc., Wilmington, DE, USA). First-strand cDNA was synthesized from 2 μg of total RNA using Superscript II (Invitrogen, Burlington, ON, Canada) and Oligo(dT)_18 _primers (Fermentas, Burlington, ON, Canada). PCR primers were designed using PrimerSelect (DNAStar Inc.) or Primer 3 (v0.4.0,  and are listed in additional file 5. PCR was conducted in a 50-μL final volume including 0.5 μL of cDNA template, 1× *Pfx *buffer, 200 μM deoxynucleotide triphosphates (dNTPs) (Fermentas), 400 nM of each primer, and 2 units of Platinum *Pfx *polymerase (Invitrogen). The PCR conditions included an initial denaturation at 94°C for 2 min, followed by 35 cycles of 94°C for 30 s, 50°C for 30 s, 68°C for 1 min per kb, with a final extension at 68°C for 5 min. PCR products were gel purified using the QIAquick gel extraction kit (Qiagen) and cloned into pJET1.2 vector supplied with the CloneJET PCR cloning kit (Fermentas) and sequenced from the two ends using BigDye reagent on an ABI3700 sequencer (Applied Biosystems, Foster city, CA, USA).

For rapid amplification of cDNA ends (3'RACE), first-strand cDNA was made from 2 μg of total RNA extracted from wild-type canola (cv. Westar) using Superscript II and an oligo(dT)_17 _adaptor sequence [125], and 0.5 μL of cDNA template was used for 3' RACE. Reactions were conducted in a 50-μL final volume including 1× Taq buffer, 0.2 mM dNTPs, 0.4 μM of each primer, and 0.2 μL (1 unit) of Platinum *Taq *polymerase (Invitrogen). The primers designed by PrimerSelect (DNAstar) are outlined in the additional file 5 and the adaptor sequence was 5'-GACTCGAGCGACATCGAT-3' [125]. The PCR conditions included an initial denaturation of 94°C for 2 min, followed by 35 cycles of 94°C for 30 s, 50°C for 30 s, 72°C for 1 min, with a final extension at 72°C for 5 min. PCR products were purified and cloned into pGEM-T vector (Promega, Madison, WI, USA) or pJET1.2 vector (Fermentas) and sequenced. Sequences were analyzed and translated using DNAStar. Based on the sequenced cDNA sequences from 3'RACE, new primers were designed, which were then used to clone the full-length cDNAs of some *BnWRKY *genes. At least two independent clones were sequenced from both ends.

### Phylogenetic tree construction and bioinformatics

The WRKY domain boundary was defined as previously described [11]. The peptide sequences of the domains were aligned using ClustalX (v1.83) with a gap opening penalty of 35 and gap extension penalty of 0.75 in pairwise alignment, and a gap opening penalty of 15 and gap extension penalty of 0.30 in multiple alignment parameters settings. The multiple alignments were adjusted with gaps manually inserted for optimal alignment based on the conserved features of the WRKY domains. The maximum parsimony algorithm implemented in MEGA4 [126] for amino acid sequences were used for phylogenetic tree reconstruction according to [127,128]. One hundred bootstrapped data sets were used to estimate the confidence of each tree clade. The protein sequences of *Arabidopsis *WRKY TFs were retrieved from TAIR  and rice WRKY TFs from the Database of Rice Transcription Factors (DRTF, ). The nomenclature of rice (*Oryza sativa*. cv japonica) WRKY TFs was as previously described [27]. Putative orthologs of *BnWRKY *genes were identified in both *Arabidopsis *and rice using the translated amino acid sequences in InParanoid [75].

### Subcellular localization and confocal microscopy

The coding regions (CDS) of *BnWRKY6*, *BnWRKY25, BnWRKY33*, and *BnWRKY75 *were amplified by RT-PCR from canola (Westar) cDNAs using the primers listed in additional file 5. PCR products were purified using a QIAquick PCR purification kit (Qiagen), restricted by *Nco *I (New England Biolabs, Ipswich, MA, USA) and/or *Bsp *HI (Fermentas), purified again and cloned into *Nco *I digested pCsGFPBT (GenBank: DQ370426) vector with a Gly-Ala- rich peptide linker between CDSs and sGFP. All constructs were sequenced and mobilized into *Agrobacterium tumefaciens *GV3101 through the freeze-thaw method and transformed into wild-type *A*. *thaliana *(Col-0) employing the floral dip method (Clough and Bent, 1998). Resistant lines were selected on 1/2 × MS containing 1% (w/v) sucrose and 50 mg/L hygromycin B (Sigma-Aldrich) for 7 d before being transferred into soil to grow the plants to maturity and to harvest T_2 _seeds, which were further sown on the same type of hygromycin-containing medium. Five-day-old seedlings from ten independent T_2 _lines were mounted on slides for GFP observation under confocal microscope (Carl Zeiss). At least five cells were screened for each line.

### Fungal pathogen inoculation and hormone treatments

Wild type canola (cv. Westar) plants were grown as described previously in a greenhouse for 18 days. Potato dextrose agar (PDA) agar plugs of *S. sclerotiorum *were prepared as described earlier [5] and placed on the first and second true leaves, which were wounded slightly. The preparation of spores of *A. brassicae *and inoculation of canola leaves were performed as described previously [4]. Leaves of uninoculated/mock plants were treated similarly with PDA agar plugs without the mycelia or with water in the case of *A. brassicae*. Plants were placed in a humidity chamber for 24 h before being placed in the greenhouse. Tissues were harvested 12, 24, 48 and 72 h post inoculation and kept at -80°C after being flash-frozen in liquid nitrogen. JA, SA, BAP and ABA were applied by spraying 50 μM JA, 1 mM SA, 20 μM BAP or 50 μM (±)-ABA (Sigma-Aldrich, St. Louis, MO, USA). A stock solution (500 μM) of JA in water was first prepared and then diluted with 0.1% (v/v) ethanol to 50 μM. ABA was first dissolved in absolute ethanol to prepare a 20 mM stock solution and then diluted with 0.1% (v/v) ethanol to the final 50 μM solution. SA was dissolved in water to prepare a 100 mM stock solution with the adjustment of pH to 6.5 using 1 M KOH before dilution in water to the 1 mM working solution and BAP was dissolved in 1 M NaOH to prepare a 1 mM stock solution after which it was diluted with water to the 20 μM working solution. The mock treatments were 0.1% (v/v) ethanol for JA and ABA, water adjusted to pH 6.5 with 1 M KOH or 1 M NaOH for SA or BAP treatments, respectively. Ethylene treatment was carried out in an airtight clear acrylic chamber (1.5 m × 0.6 m × 0.6 m) placed in the same greenhouse, into which 100 ppm ethylene gas in air (Praxair, Mississauga, ON, Canada) was passed at a rate of 2 L/min. Mock plants were placed in a separate chamber into which air (Praxair) was passed at the same rate. Leaves from mock and hormone treated plants were harvested at 6 and 24 h time points, flash frozen in liquid nitrogen and stored at -80°C. The entire sample preparation was independently repeated three times.

### Quantitative RT-PCR (qRT-PCR)

Total RNA was isolated from mock, inoculated or hormone treated leaf tissue using the RNeasy Plant Mini kit (Qiagen) with on-column DNA digestion. RNA was quantified by NanoDrop ND-1000 (NanoDrop Technologies, Inc.) and the integrity of the RNA was assessed on a 1% (w/v) agarose gel. Primers were designed using PrimerExpress3.0 (Applied Biosystems) targeting an amplicon size of 80–150 bp. The primers used are listed in the additional file 5. The specificity of all primers designed was submitted to BLASTn search against NCBI *B. napus *nr and EST databases and any nonspecific primers were eliminated or redesigned. Hence, the results from qRT-PCR analysis might represent the response of specific *BnWRKY *genes. The qRT-PCR assay was performed as described previously [5]. qRT-PCR for each gene was performed in duplicate for each of the three independent biological replicates. Significance was determined with SAS software version 9.1 (SAS Institute Inc.) (*p *value < 0.05).

### Accession numbers

The cDNA sequences of 38 BnWRKY TF genes cloned in this study were deposited in public database [GenBank: EU912389–EU912407, EU912409–EU912418, FJ012166–FJ012171, FJ210288–FJ210290 and FJ384101–FJ384114].

## Authors' contributions

BY designed, carried out all the experiments and drafted the manuscript. YQJ participated in data analysis, and confocal microscopy. MHR provided assistance. NNVK and MKD provided research facility/tools. NNVK designed and supervised the research. All authors contributed to the writing and editing of the manuscript and approved the manuscript.

## Supplementary Material

Additional file 1 **Supplementary Table 1**. B. napus (canola) WRKY transcription factors identified in this study. Click here for file

Additional file 2 **Supplementary table 2**. Expression sequence tags (ESTs) identified for *BnWRKY *genes. Click here for file

Additional file 3 **Alignment of Sequences of 53 WRKY domains of BnWRKY transcription factors**. Identical amino acids are shaded in black, and similar amino acids are shaded in gray. The conserved WRKYGQK heptapeptide or its variants are underlined at the top of the alignment and, the cysteines and histidines of the C2H2- or C2HC-type zinc finger motif are indicated by arrows. The consensus amino acids are shown at the bottom of the alignment. This alignment was produced by BOXSHADE 3.21 . Click here for file

Additional file 4 **A bootstrap consensus maximum parsimony tree of WRKY TFs in canola, Arabidopsis and rice (*japonica*)**. Only the WRKY domain residues were aligned using ClustalX (v1.83) and the evolutionary history was inferred using the maximum parsimony method in MEAG4. The percentage of replicate trees is shown on the branches and it is calculated in the bootstrap test (500 replicates) for the associated taxa being clustered together. All alignment gaps were treated as missing data. There were a total of 150 positions in the final dataset, out of which 66 were parsimony informative. The two letters N and C after group I represents the N-terminal and the C-terminal WRKY domains of group I proteins, respectively. A chlorophyte alga, *Ostreococcus tauri *(Ot) WRKY (Acc. CAL54953) is used as the outgroup. Click here for file

Additional file 5 **Primers used in this study**. F, forward primer for RT-PCR; R, reverse primer for RT-PCR; QF, qRT-PCR forward primer; QR, qRT-PCR reverse primer; RACE-F, 3'RACE forward primer; GFP-F, forward primer for N-terminal GFP fusion; GFP-R, reverse primer for N-terminal GFP fusion. Click here for file
