# Supplementary figures and images for: Identification and expression analysis of WRKY transcription factor genes in canola (Brassica napus L.) in response to fungal pathogens and hormone treatments
Source: BMC Plant Biol. 2009 Jun 3;9:68. doi: 10.1186/1471-2229-9-68 (PMC2698848; doi:10.1186/1471-2229-9-68)

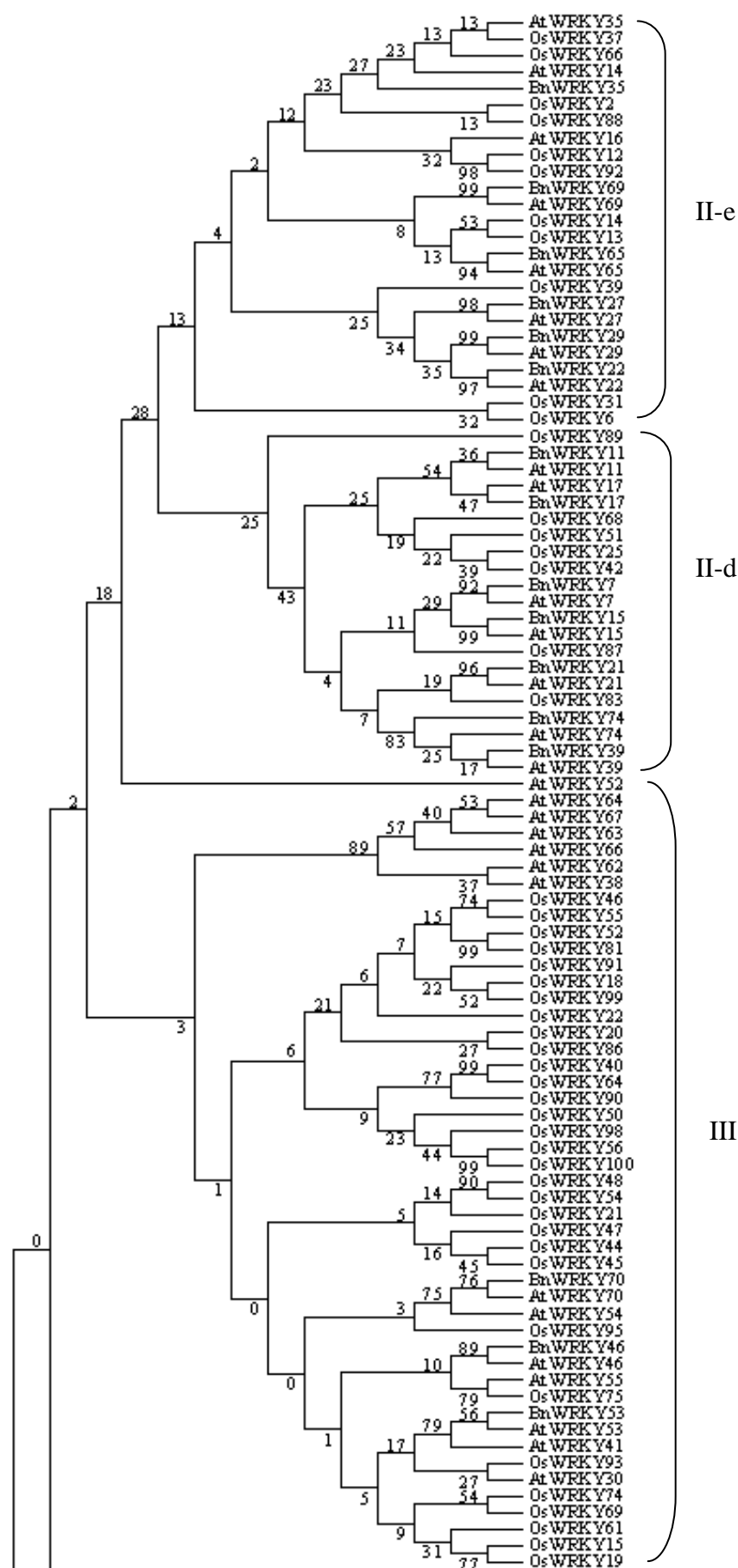

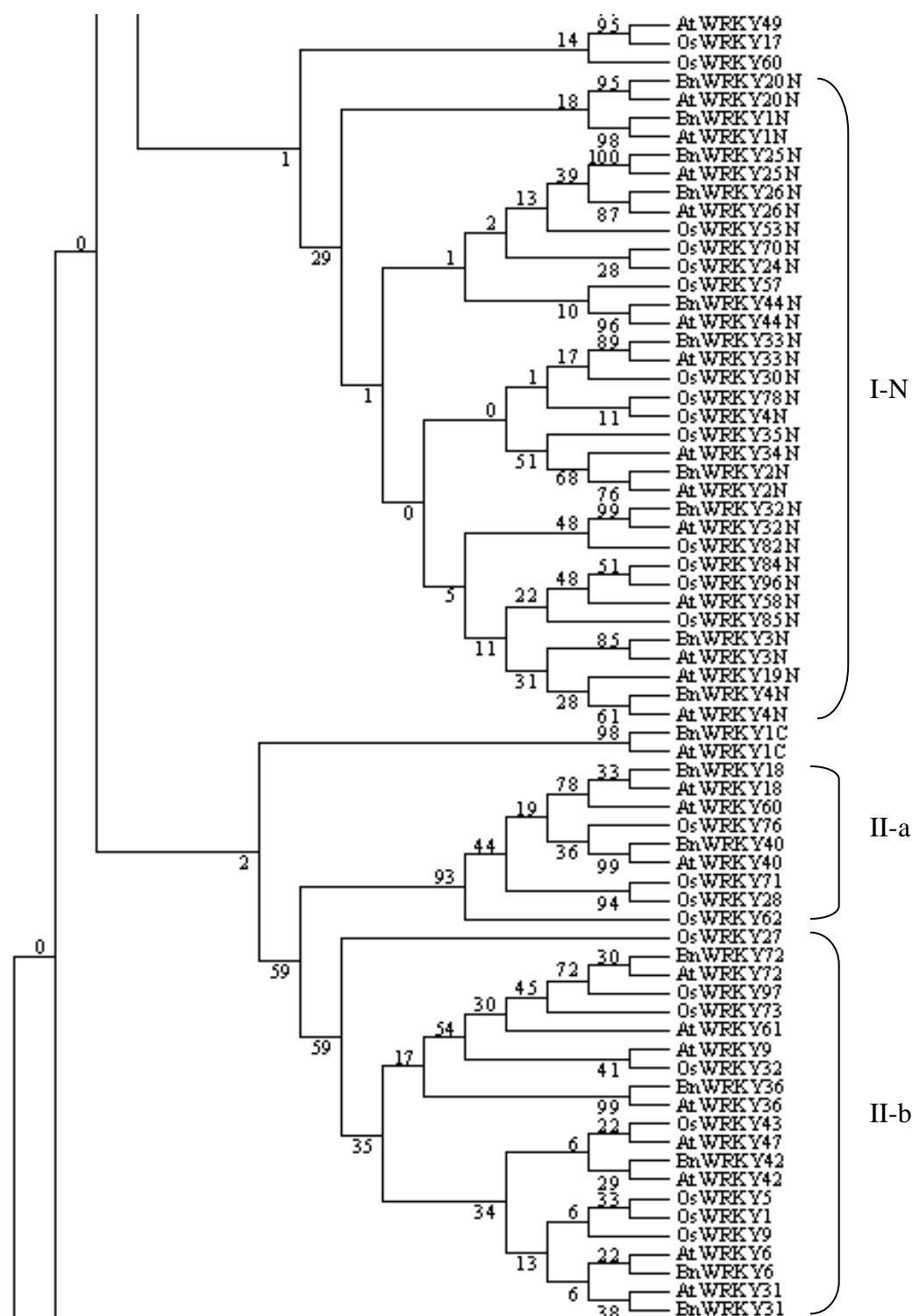

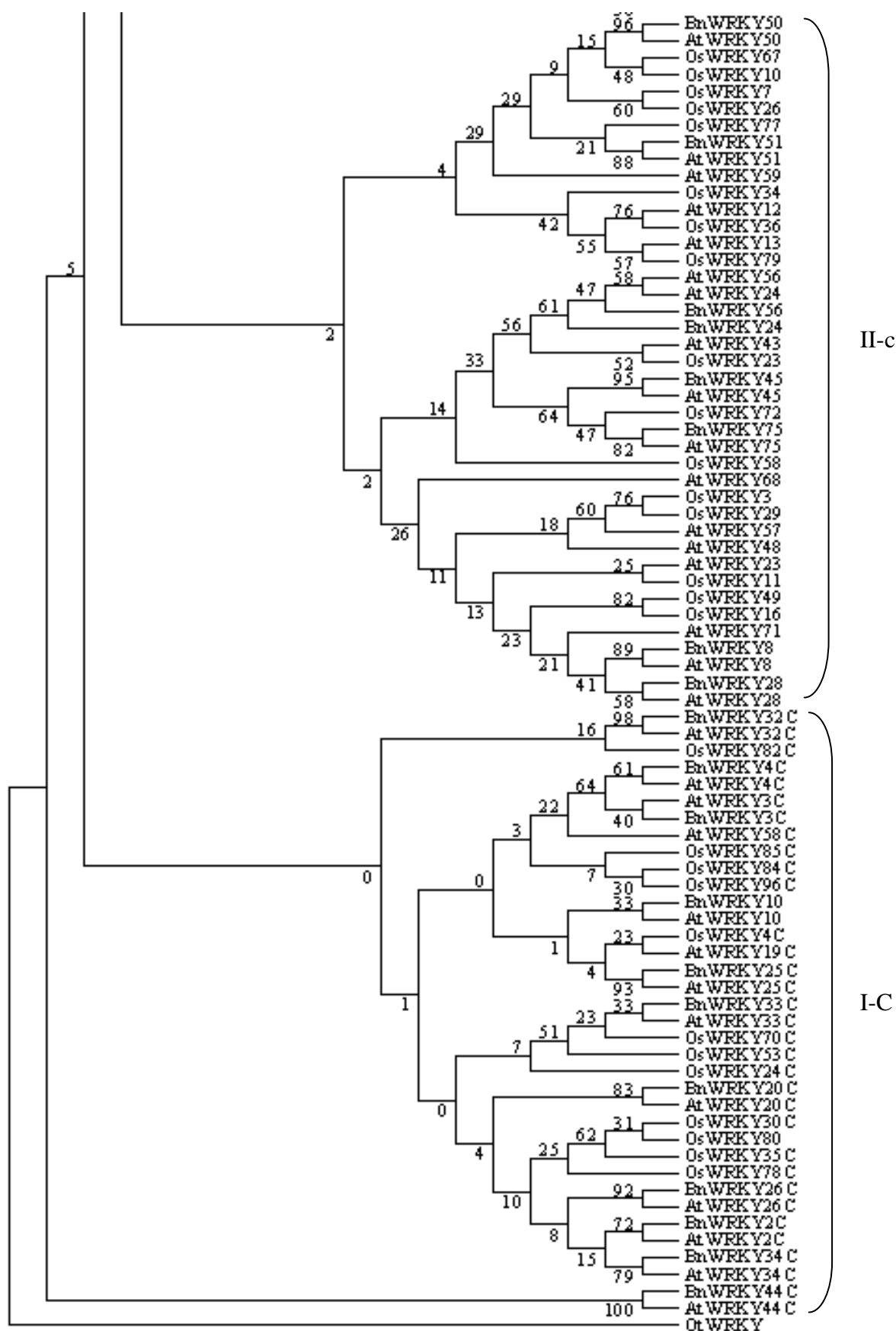

Supplement: Additional file 4 — A bootstrap consensus maximum parsimony tree of WRKY TFs in canola, Arabidopsis and rice (japonica). Only the WRKY domain residues were aligned using ClustalX (v1.83) and the evolutionary history was inferred using the maximum parsimony method in MEAG4. The percentage of replicate trees is shown on the branches and it is calculated in the bootstrap test (500 replicates) for the associated taxa being clustered together. All alignment gaps were treated as missing data. There were a total of 150 positions in the final dataset, out of which 66 were parsimony informative. The two letters N and C after group I represents the N-terminal and the C-terminal WRKY domains of group I proteins, respectively. A chlorophyte alga, Ostreococcus tauri (Ot) WRKY (Acc. CAL54953) is used as the outgroup. [file 1471-2229-9-68-S4.pdf]
