# Supplementary material for: Identification and expression analysis of WRKY transcription factor genes in canola (Brassica napus L.) in response to fungal pathogens and hormone treatments
Source: BMC Plant Biol. 2009 Jun 3;9:68. doi: 10.1186/1471-2229-9-68 (PMC2698848; doi:10.1186/1471-2229-9-68)
Supplement: Additional file 3 — Alignment of Sequences of 53 WRKY domains of BnWRKY transcription factors. Identical amino acids are shaded in black, and similar amino acids are shaded in gray. The conserved WRKYGQK heptapeptide or its variants are underlined at the top of the alignment and, the cysteines and histidines of the C2H2- or C2HC-type zinc finger motif are indicated by arrows. The consensus amino acids are shown at the bottom of the alignment. This alignment was produced by BOXSHADE 3.21 . [file 1471-2229-9-68-S3.pdf]

```
BnWRKY4N    1 NVDKPADDGYNWRKYGQKQVKGSEFP-------------------------RSYYKCTNPG---CPVKKKVER-SLDG-QVTEIIYKGQHNHEPP
BnWRKY3N    1 NADKPADDGYNWRKYGQKQVKGSDFP-------------------------RSYYKCTHPA---CPVKKKVER-SQDG-QVTEIIYKGQHSHEPP
BnWRKY20N   1 APAVLADDGYNWRKYGQKHVKGSEFP-------------------------RSYYKCTHPN---CEVKKLFER-SYDG-QITDIIYKGTHDHPKP
BnWRKY2N    1 VGGAPAEDGYNWRKYGQKLVKGSEYP-------------------------RSYYKCTNPN---CPVKKKVER-SREG-HITEIIYKGAHNHSKP
BnWRKY33N   1 REQRKGEDGYNWRKYGQKQVKGSENP-------------------------RSYYKCTFPS---CPTKKKVER-SLEG-QITEIVYKGSHNHPKP
BnWRKY26N   1 SGNKTSEDGYNWRKYGQKQVKGSENP-------------------------RSYFRCTYPN---CLTKKKVET-SLVKGHVTEIVYKGSHNHPKP
BnWRKY25N   1 MVSRNSNDGYGWRKYGQKQVKKSENP-------------------------RSYFKCTYPN---CVSKKIVET-TSDG-QITEIIYKGGHNHPKP
BnWRKY44N   1 AGDRSSVDGYNWRKYGQKQVKGSDCP-------------------------RSYYKCTHPK---CPVKKKVER-SMGG-LVSEIVYQGEHNHSKP
BnWRKY1N    1 IREKVMEDGYNWRKYGQKLVKGNEFV-------------------------RSYYRCTHPN---CKAKKQLER-SPGG-QIVDTVYFGEHDHPKP
BnWRKY32N   1 VLRTPAHDGYHWRKYGQKQVKSPKGS-------------------------RSYYRCTYSD---CCAKKIECS-NDSG-NVIEIVNKGSHSHEPL
BnWRKY6     1 SEASMISDGCQWRKYGQKMAKGNPCP-------------------------RAYYRCTMAT--GCPVRKQVQRCAED-RSILITTYEGNHNHPLP
BnWRKY31    1 --ATMITDGCQWRKYGQKMAKGNPCP-------------------------RAYYRCTMAG--GCPVRKQVQRCAED-RSILITTYEGNHNHPLP
BnWRKY42    1 SEAPTLSDGCHWRKYGQKMAKGNPCP-------------------------RAYFRCTMAV--GCPVRKQVQRCAEE-RSILITTYEGNHNHPLP
BnWRKY72    1 CDTPTMNDGCQWRKYGQKIAKGNPCP-------------------------RAYYRCTVAP--GCPVRKQVQRCADD-MSILITTYEGTHSHPLP
BnWRKY36    1 CEDPSINDGCQWRKYGQKTAKANPLP-------------------------RAYYRCSMSS--NCPVRKQVQRCGEDDTSAYMTTYEGTHDHPLP
BnWRKY18    1 DTSLTVKDGYQWRKYGQKVTRDNPSP-------------------------RAYFRCSFAP--SCPVKKKVQRSAED-PSILVATYEGTHNHLGP
BnWRKY40    1 DTTLVVKDGYQWRKYGQKVTRDNPSP-------------------------RAYFKCACAP--SCSVKKKVQRSVED-QSVLVATYEGEHNHPMP
BnWRKY11    1 KIADIPPDEFSWRKYGQKPIKGSPHP-------------------------RGYYKCSTFR--GCPARKHVERALDD-PAMLIVTYEGEHHHKQS
BnWRKY17    1 KIADIPADEYSWRKYGQKPIKGSPHP-------------------------RGYYKCSTFR--GCPARKHVERALDD-PTMLIVTYEGEHRHQKS
BnWRKY74    1 KIADIPPDEYSWRKYGQKPIKGSPHP-------------------------RGYYKCSSVR--GCPARKHVERCVDE-TSMLIVTYEGEHNHARI
BnWRKY39    1 KIADIPPDEYSWRKYGQKPIKGSPHP-------------------------RGYYKCSSVR--GCPARKHVERCVDE-TSMLIVTYEGEHNHSRI
BnWRKY7     1 KMADIPSDEFSWRKYGQKPIKGSPHP-------------------------RGYYKCSSMR--GCPARKHVERAPDD-AMMLIVTYEGDHNHAMV
BnWRKY15    1 KMSDVPPDDYSWRKYGQKPIKGSPHP-------------------------RGYYKCSSVR--GCPARKHVERAADD-SSMLIVTYEGDHNHSLS
BnWRKY21    1 KVADIPPDDYSWRKYGQKPIKGSPYP-------------------------RGYYKCSSMR--GCPARKHVERCVED-PAMLIVTYEAEHSHPKL
BnWRKY27    1 TQENLSSDMWAWRKYGQKPIKGSPYP-------------------------RNYYRCSSSK--GCLARKQVERSNLD-PNIFIVTYTGEHTHPRP
BnWRKY29    1 KEENLLSDAWAWRKYGQKPIKGSPYP-------------------------RSYYRCSSSK--GCLARKQVERNPQN-PEKFTITYTNEHNHELP
BnWRKY22    1 AAEALNNDVWAWRKYGQKPIKGSPYP-------------------------RGYYRCSTSK--GCLARKQVERNRSD-PTMFIVTYTAEHNHPAP
BnWRKY35    1 SGEVVPSDLWAWRKYGQKPIKGLPYP-------------------------RGYYRCSSSK--GCPARKQVERSRTD-PNMLVITYTSEHNHPWP
BnWRKY65    1 GDTTPPSDSWAWRKYGQKPIKGSPYP-------------------------RGYYRCSSTK--GCPARKQVERSRDD-PTMIIITYTSEHNHPWP
BnWRKY69    1 GEVYPPSDSWAWRKYGQKPIKGSPYP-------------------------RGYYRCSSSK--GCPARKQVERSRVD-PSKLMITYACDHNHPFP
BnWRKY8     1 TEVDHLEDGYRWRKYGQKAVKNSPYP-------------------------RSYYRCTTQ---KCNVKKRVERSYQD-PTVVITTYESQHDHPIP
BnWRKY28    1 SEVDHLEDGYRWRKYGQKAVKNSPYP-------------------------RSYYRCTTQ---KCNVKKRVERSFQD-PTVVITTYEGQHNHPIP
BnWRKY24    1 SDDDVLDDGYRWRKYGQKSVKNNGHP-------------------------RSYYRCTYH---TCNVKKQVQRLAKD-PKIIVTTYEGIHNHPCE
BnWRKY56    1 -DDDVLDDGYRWRKYGQKSVKNNAHP-------------------------RSYYRCTYH---TCNVKKQVQRLAKD-PKVVVTTYEGVHNHPCE
BnWRKY75    1 SQVDILDDGYRWRKYGQKAVKNNTFP-------------------------RSYYRCTYA---GCNVKKQVQRLTSD-QEVVVTTYEGVHSHAIE
BnWRKY45    1 SQVDILDDGYRWRKYGQKAVKNNPFP-------------------------RSYYKCTEK---GCRVKKQVQRLSGD-EGVVVTTYQGVHTHPVD
BnWRKY50    1 SDVEVLDDGFKWRKYGKKMVKNSPHP-------------------------RNYYKCAAD---ACPVKKRVERDKDD-PSFVITTYEGSHNHSSM
BnWRKY20C   1 SEVDILDDGYRWRKYGQKVVRGNPNP-------------------------RSYYKCTAP---GCPVRKHVERASHD-PKAVITTYEGKHNHDVP
BnWRKY2C    1 SDVDILDDGYRWRKYGQKVVKGNPNP-------------------------RSYYKCTAP---GCTVRKHVERASHD-LKSVITTYEGKHNHDVP
BnWRKY33C   1 SDIDILDDGYRWRKYGQKVVKGNPNP-------------------------RSYYKCTTI---GCPVRKHVERASQD-LRAVITTYEGKHNHDVP
BnWRKY26C   1 SDIDILDDGYRWRKYGQKVVKGNPNPRFCFFFCSQLQLQGGAYLPKTCDARRSYYKCTFT---GCCVRKQVERAFHD-AKSVITTYEGKHNHQIP
BnWRKY34C   1 TDVDILEDGYRWRKYGQKVVKGNPNP-------------------------RSYYKCTAT---GCIVTKHVERASDD-LTSVLTTYIGKHTHEVP
BnWRKY4C    1 SEVDLLDDGYRWRKYGQKVVKGNPYP-------------------------RSYYKCTTQ---GCGVRKHVERAATD-PKAVVTTYEGKHNHDLP
BnWRKY3C    1 SEVDLLDDGFRWRKYGQKVVKGNPYP-------------------------RSYYKCTTP---GCGVRKHVERAAND-PKAVVTTYEGKHNHDVP
BnWRKY25C   1 SEIDVLIDGFRWRKYGQKVVKGNTNP-------------------------RSYYKCTYQ---GCGVRKQVERSAED-ERAVLTTYEGRHNHDVP
BnWRKY44C   1 VESDSLEDGFRWRKYGQKVVGGNAHP-------------------------RSYYRCTSA---NCRARKHVERASDD-PRAFITTYEGKHNHHLN
BnWRKY1C    1 TLFDIVNDGYRWRKYGQKSVKGSPYP-------------------------RSYYRCSSS---GCPVKKHVERSSRD-TKMLIMTYEGNHDHDMP
BnWRKY10    1 -------------------------C-------------------------RSYYKCTYT---ACDVKKHVERGAED-VKFLLVTYDGIHEHDPP
BnWRKY32C   1 GDVGISGDGYRWRKYGQKMVKGNPNP-------------------------RNYYRCTSA---GCPVRKHIETSGEN-KTAVVITYKGVHNHDMP
BnWRKY53    1 GLEGPQDDVYSWRKYGQKDILGAKFP-------------------------RSYYRCTHRSTQNCWATKQVQRSDSD-PTVFELTYRGTHTCRQG
BnWRKY70    1 VESTVLDDAFSWRKYGQKEILNAIFP-------------------------RSYFRCTHKFTQGCKATKQVQKLEPE-SKMFNITYIGNHTCNTK
BnWRKY46    1 QEGTPLDDGHCWRKYGQKDIHGSKNP-------------------------RGYYRCTHRFTRGCLVVKQVQKSDIN-PMCYEVKYVESHTCDIT
BnWRKY51    1 SKIDVMDDGYKWRKYGRKSGKNNSNK-------------------------REYYQCLNER----LTGEKKVRDGED-ATYVITTYDGGHNHESA
consensus   1     lm dgy wrkygqk vkg p p                         RsYykCt     gc vrkkver   d   vviityeg Hnh  p
```

```
BnWRKY4N     1 NVDKPADDGYNWRKYGQKQVKGSEFP-------------------------RSYYKCTNPG---CPVKKKVER-SLDG-QVTEIIYKGQHNHEPP
BnWRKY3N     1 NADKPADDGYNWRKYGQKQVKGSDFP-------------------------RSYYKCTHPA---CPVKKKVER-SQDG-QVTEIIYKGQHSHEPP
BnWRKY20N    1 APAVLADDGYNWRKYGQKHVKGSEFP-------------------------RSYYKCTHPN---CEVKKLFER-SYDG-QITDIIYKGTHDHPKP
BnWRKY2N     1 VGGAPAEDGYNWRKYGQKLVKGSEYP-------------------------RSYYKCTNPN---CPVKKKVER-SREG-HITEIIYKGAHNHSKP
BnWRKY33N    1 REQRKGEDGYNWRKYGQKQVKGSENP-------------------------RSYYKCTFPS---CPTKKKVER-SLEG-QITEIVYKGSHNHPKP
BnWRKY26N    1 SGNKTSEDGYNWRKYGQKQVKGSENP-------------------------RSYFKCTYPN---CLTKKKVET-SLVKGHVTEIVYKGSHNHPKP
BnWRKY25N    1 MVSRNSNDGYGWRKYGQKQVKKSENP-------------------------RSYFKCTYPN---CVSKKIVET-TSDG-QITEIIYKGGHNHPKP
BnWRKY44N    1 AGDRSSVDGYNWRKYGQKQVKGSDCP-------------------------RSYYKCTHPK---CPVKKKVER-SMGG-LVSEIVYQGEHNHSKP
BnWRKY1N     1 IREKVMEDGYNWRKYGQKLVKGNEFV-------------------------RSYYRCTHPN---CKAKKQLER-SPGG-QIVDTVYFGEHDHPKP
BnWRKY32N    1 VLRTPAHDGYHWRKYGQKQVKSPKGS-------------------------RSYYRCTYSD---CCAKKIECS-NDSG-NVIEIVNKGSHSHEPL
BnWRKY6      1 SEASMISDGCQWRKYGQKMAKGNPCP-------------------------RAYYRCTMAT--GCPVRKQVQRCAED-RSILITTYEGNHNHPLP
BnWRKY31     1 --ATMITDGCQWRKYGQKMAKGNPCP-------------------------RAYYRCTMAG--GCPVRKQVQRCAED-RSILITTYEGNHNHPLP
BnWRKY42     1 SEAPTLSDGCHWRKYGQKMAKGNPCP-------------------------RAYFRCTMAV--GCPVRKQVQRCAEE-RSILITTYEGNHNHPLP
BnWRKY72     1 CDTPTMNDGCQWRKYGQKIAKGNPCP-------------------------RAYYRCTVAP--GCPVRKQVQRCADD-MSILITTYEGTHSHPLP
BnWRKY36     1 CEDPSINDGCQWRKYGQKTAKANPLP-------------------------RAYYRCSMSS--NCPVRKQVQRCGEDDTSAYMTTYEGTHDHPLP
BnWRKY18     1 DTSLTVKDGYQWRKYGQKVTRDNPSP-------------------------RAYFRCSFAP--SCPVKKKVQRSAED-PSILVATYEGTHNHLGP
BnWRKY40     1 DTTLVVKDGYQWRKYGQKVTRDNPSP-------------------------RAYFKCACAP--SCSVKKKVQRSVED-QSVLVATYEGEHNHPMP
BnWRKY11     1 KIADIPPDEFSWRKYGQKPIKGSPHP-------------------------RGYYKCSTFR--GCPARKHVERALDD-PAMLIVTYEGEHHHKQS
BnWRKY17     1 KIADIPADEYSWRKYGQKPIKGSPHP-------------------------RGYYKCSTFR--GCPARKHVERALDD-PTMLIVTYEGEHRHQKS
BnWRKY74     1 KIADIPPDEYSWRKYGQKPIKGSPHP-------------------------RGYYKCSSVR--GCPARKHVERCVDE-TSMLIVTYEGEHNHARI
BnWRKY39     1 KIADIPPDEYSWRKYGQKPIKGSPHP-------------------------RGYYKCSSVR--GCPARKHVERCVDE-TSMLIVTYEGEHNHSRI
BnWRKY7      1 KMADIPSDEFSWRKYGQKPIKGSPHP-------------------------RGYYKCSSMR--GCPARKHVERAPDD-AMMLIVTYEGDHNHAMV
BnWRKY15     1 KMSDVPPDDYSWRKYGQKPIKGSPHP-------------------------RGYYKCSSVR--GCPARKHVERAADD-SSMLIVTYEGDHNHSLS
BnWRKY21     1 KVADIPPDDYSWRKYGQKPIKGSPYP-------------------------RGYYKCSSMR--GCPARKHVERCVED-PAMLIVTYEAEHSHPKL
BnWRKY27     1 TQENLSSDMWAWRKYGQKPIKGSPYP-------------------------RNYYRCSSSK--GCLARKQVERSNLD-PNIFIVTYTGEHTHPRP
BnWRKY29     1 KEENLLSDAWAWRKYGQKPIKGSPYP-------------------------RSYYRCSSSK--GCLARKQVERNPQN-PEKFTITYTNEHNHELP
BnWRKY22     1 AAEALNNDVWAWRKYGQKPIKGSPYP-------------------------RGYYRCSTSK--GCLARKQVERNRSD-PTMFIVTYTAEHNHPAP
BnWRKY35     1 SGEVVPSDLWAWRKYGQKPIKGLPYP-------------------------RGYYRCSSSK--GCPARKQVERSRTD-PNMLVITYTSEHNHPWP
BnWRKY65     1 GDTTPPSDSWAWRKYGQKPIKGSPYP-------------------------RGYYRCSSTK--GCPARKQVERSRDD-PTMIIITYTSEHNHPWP
BnWRKY69     1 GEVYPPSDSWAWRKYGQKPIKGSPYP-------------------------RGYYRCSSSK--GCPARKQVERSRVD-PSKLMITYACDHNHPFP
BnWRKY8      1 TEVDHLEDGYRWRKYGQKAVKNSPYP-------------------------RSYYRCTTQ---KCNVKKRVERSYQD-PTVVITTYESQHDHPIP
BnWRKY28     1 SEVDHLEDGYRWRKYGQKAVKNSPYP-------------------------RSYYRCTTQ---KCNVKKRVERSFQD-PTVVITTYEGQHNHPIP
BnWRKY24     1 SDDDVLDDGYRWRKYGQKSVKNNGHP-------------------------RSYYRCTYH---TCNVKKQVQRLAKD-PKIIVTTYEGIHNHPCE
BnWRKY56     1 -DDDVLDDGYRWRKYGQKSVKNNAHP-------------------------RSYYRCTYH---TCNVKKQVQRLAKD-PKVVVTTYEGVHNHPCE
BnWRKY75     1 SQVDILDDGYRWRKYGQKAVKNNTFP-------------------------RSYYRCTYA---GCNVKKQVQRLTSD-QEVVVTTYEGVHSHAIE
BnWRKY45     1 SQVDILDDGYRWRKYGQKAVKNNPFP-------------------------RSYYKCTEK---GCRVKKQVQRLSGD-EGVVVTTYQGVHTHPVD
BnWRKY50     1 SDVEVLDDGFKWRKYGKKMVKNSPHP-------------------------RNYYKCAAD---ACPVKKRVERDKDD-PSFVITTYEGSHNHSSM
BnWRKY20C    1 SEVDILDDGYRWRKYGQKVVRGNPNP-------------------------RSYYKCTAP---GCPVRKHVERASHD-PKAVITTYEGKHNHDVP
BnWRKY2C     1 SDVDILDDGYRWRKYGQKVVKGNPNP-------------------------RSYYKCTAP---GCTVRKHVERASHD-LKSVITTYEGKHNHDVP
BnWRKY33C    1 SDIDILDDGYRWRKYGQKVVKGNPNP-------------------------RSYYKCTTI---GCPVRKHVERASQD-LRAVITTYEGKHNHDVP
BnWRKY26C    1 SDIDILDDGYRWRKYGQKVVKGNPNPRFCFFFCSQLQLQGGAYLPKTCDARRSYYKCTFT---GCCVRKQVERAFHD-AKSVITTYEGKHNHQIP
BnWRKY34C    1 TDVDILEDGYRWRKYGQKVVKGNPNP-------------------------RSYYKCTAT---GCIVTKHVERASDD-LTSVLTTYIGKHTHEVP
BnWRKY4C     1 SEVDLLDDGYRWRKYGQKVVKGNPYP-------------------------RSYYKCTTQ---GCGVRKHVERAATD-PKAVVTTYEGKHNHDLP
BnWRKY3C     1 SEVDLLDDGFRWRKYGQKVVKGNPYP-------------------------RSYYKCTTP---GCGVRKHVERAAND-PKAVVTTYEGKHNHDVP
BnWRKY25C    1 SEIDVLIDGFRWRKYGQKVVKGNTNP-------------------------RSYYKCTYQ---GCGVRKQVERSAED-ERAVLTTYEGRHNHDVP
BnWRKY44C    1 VESDSLEDGFRWRKYGQKVVGGNAHP-------------------------RSYYRCTSA---NCRARKHVERASDD-PRAFITTYEGKHNHHLN
BnWRKY1C     1 TLFDIVNDGYRWRKYGQKSVKGSPYP-------------------------RSYYRCSSS---GCPVKKHVERSSRD-TKMLIMTYEGNHDHDMP
BnWRKY10     1 -------------------------C-------------------------RSYYKCTYT---ACDVKKHVERGAED-VKFLLVTYDGIHEHDPP
BnWRKY32C    1 GDVGISGDGYRWRKYGQKMVKGNPNP-------------------------RNYYRCTSA---GCPVRKHIETSGEN-KTAVVITYKGVHNHDMP
BnWRKY53     1 GLEGPQDDVYSWRKYGQKDILGAKFP-------------------------RSYYRCTHRSTQNCWATKQVQRSDSD-PTVFELTYRGTHTCRQG
BnWRKY70     1 VESTVLDDAFSWRKYGQKEILNAIFP-------------------------RSYFRCTHKFTQGCKATKQVQKLEPE-SKMFNITYIGNHTCNTK
BnWRKY46     1 QEGTPLDDGHCWRKYGQKDIHGSKNP-------------------------RGYYRCTHRFTRGCLVVKQVQKSDIN-PMCYEVKYVESHTCDIT
BnWRKY51     1 SKIDVMDDGYKWRKYGRKSGKNNSNK-------------------------REYYQCLNER----LTGEKKVRDGED-ATYVITTYDGGHNHESA
consensus    1     lm dgy wrkygqk vkg p p                         RsYykCt     gc vrkkver   d   vviityeg Hnh  p
```
